# Recent Advances and Critical Review on Two-Dimensional Black Phosphorus: Preparation and Optoelectronic Applications

**DOI:** 10.3390/ma19132691

**Published:** 2026-06-23

**Authors:** Jialu Zheng, Zeying Zhou, Danghui Wang, Yan Li, Zhao Li

**Affiliations:** 1 School of Materials Science and Engineering, Xi’an Shiyou University, Xi’an 710065, China; zzy9ps@163.com (Z.Z.); li1988yan@163.com (Y.L.); lizhao@xsyu.edu.cn (Z.L.); 2 College of New Energy, Xi’an Shiyou University, Xi’an 710065, China; wdhyxp@outlook.com

**Keywords:** 2D black phosphorus, controllable fabrication, optoelectronic applications, stability

## Abstract

**Highlights:**

- Reviews state of the art progress in 2D black phosphorus research.
- Highlights Compares diverse controllable synthesis strategies for 2D black phosphorus.
- Applications in field-effect transistors and photodetectors.

**Abstract:**

Two-dimensional black phosphorus (2D BP) has emerged as one of the most promising two-dimensional semiconductors for next-generation micro and nanoelectronics beyond Moore’s Law. It is distinguished by its unique combination of a layer dependent direct bandgap, broadband photoresponse, and pronounced in-plane anisotropy, addressing key intrinsic limitations that have hindered the widespread application of graphene and conventional transition metal dichalcogenides (TMDCs). This review provides a systematic and comprehensive overview of recent advances in the controllable fabrication of 2D BP and its applications in transistors and photodetectors. We first elucidate its crystal lattice structure and fundamental physical properties, then categorize and summarize synthesis strategies based on production scale ranging from small scale methods (e.g., mechanical exfoliation and solution based exfoliation) to large scale methods (e.g., Chemical Vapor Deposition (CVD) and Pulsed Laser Deposition (PLD)), with a particular focus on recent advances in high-speed field-effect transistors and broadband photodetectors. In summary, the key to achieving large-scale controllable synthesis lies in addressing the challenges of high-temperature oxidation of black phosphorus and the uncontrollable diffusion of phosphorus sources. In the future, industrial applications are expected to be realized through CVD based regulation of phosphorus sources, low-temperature growth by PLD, and deep integration with silicon-based processes.

## 1. Introduction

Two-dimensional (2D) materials exhibit unique properties that surpass those of 3D bulk materials, including strong quantum confinement, widely tunable layer-dependent bandgaps, ultrahigh specific surface areas, excellent carrier transport, and mechanical flexibility. As microelectronics continue to advance, conventional 3D semiconductors face severe bottlenecks in aggressive device scaling, such as short-channel effects, quantum tunneling, and uncontrolled power consumption [1,2]. Developing 2D semiconductors with high mobility and appropriate bandgaps has thus become critical for overcoming device limitations in the era after Moore’s Law [3,4].

Black phosphorus (BP), an irreplaceable 2D semiconductor, has weakly bonded van der Waals layers enabling facile exfoliation into few-layer/monolayer nanosheets. It combines excellent carrier transport with a layer-tunable direct bandgap (~0.3 eV for bulk to ~1.7 eV for monolayer) [5], enabling broad-spectrum absorption from visible to mid-infrared. This compensates for graphene’s zero-bandgap limitations and addresses most TMDCs’ poor near/mid-infrared absorption, forming unique performance complementarity [6,7,8].

First synthesized in 1914 via high pressure [9], BP gained prominence in 2014 when few-layer BP was exfoliated to fabricate the first BP field-effect transistor with ~1000 cm^2^·V^−1^·s^−1^ room-temperature mobility [10]. Its puckered honeycomb lattice induces strong in-plane anisotropy [11,12], enabling novel device design while effectively suppressing short-channel effects for low-power operation.

BP shows superior potential in high-speed transistors [10], mid-infrared photodetectors [13], and optical modulators [14], despite challenges of ambient oxidative degradation and unresolved large-scale oxidation inhibition [15,16]. This review outlines the basic properties of BP, classifies its preparation strategies into three categories: small-batch high-quality device research, large-scale processing methods, and wafer-scale thin film growth, and summarizes corresponding stability regulation strategies in a dedicated section, and focuses on its representative applications in field-effect transistors and photodetectors, providing a reference for future fundamental research and industrialization.

## 2. Structure and Properties of Two-Dimensional Black Phosphorus

**Figure 1:**
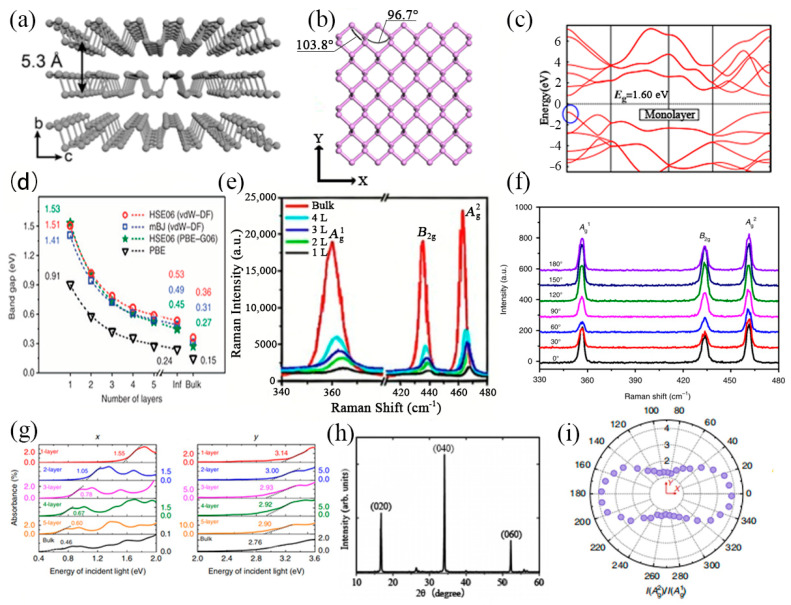
(**a**) Front view of BP [24]. Copyright 2019, Wang(s), CC BY 4.0; (**b**) Top view of monolayer BP [11]; (**c**) Band structure of the monolayer calculated using the GW method. Zero energy corresponds to the midgap. Blue circles indicate the band splitting near the band gap [20]. Copyright 2014, American Physical Society; (**d**) Direct bandgap evolution with sample thickness. Functionals used for structural optimization are shown in parentheses [22], Qiao(s), CC BY-NC-ND 4.0; (**e**) Raman spectra of 2D BP with different layer numbers and bulk BP [25]. Copyright 2015 Advanced Functional Materials; (**f**) Raman curves of BP under 632 nm laser excitation at different incident angles ranging from 0° to 180° [23]. Copyright 2019, Zhao(s), CC BY 4.0; (**g**) The optical absorption spectra of few-layer black phosphorus (BP) under two polarization directions (x and y) exhibit strong anisotropy [22], Qiao(s), CC BY-NC-ND 4.0; (**h**) X-Ray Diffraction (XRD) diffraction pattern of BP powder [26]. Copyright 2023 Crystal Research And Technology; (**i**) The ratio of the Raman $A_{g}^{2}$ and $A_{g}^{1}$ peak intensities as a function of the excitation laser polarization, exhibiting strong anisotropy [27], used under CC BY 4.0.

Black phosphorus (BP) is a layered semiconductor [17] with pronounced in-plane anisotropy along the armchair and zigzag directions. Phosphorus atoms stack in puckered six-membered rings, and layers are bonded by weak van der Waals forces [7,18,19], enabling facile exfoliation into 2D nanosheets. This provides a material foundation for fabricating high-performance polarization-sensitive photodetectors. The atomic arrangement is illustrated in Figure 1a (front view) and Figure 1b (top view of monolayer BP).

Its electronic band structure (Figure 1c) shows that both conduction band and valence band edges lie at the Γ point of the Brillouin zone, confirming it as a direct bandgap semiconductor. According to carrier effective mass theory, higher band curvature corresponds to smaller electron effective mass and higher carrier mobility, while flatter bands yield the opposite. Distinct band curvature differences exist along high-symmetry paths in different crystallographic directions: band curvatures near the conduction band minimum and valence band maximum along the armchair direction are much larger than those along the zigzag direction, causing order-of-magnitude differences in electron and hole effective masses—the central physical origin of its anisotropic electrical and optoelectronic properties. Blue circles in Figure 1c indicate the band splitting near the band gap [20]. BP exhibits a strong layer-dependent bandgap. It has been reported [21] that on sapphire substrates, monolayer BP has a bandgap of 1.73 eV (visible-near infrared response), bilayer 1.15 eV, trilayer 0.83 eV, and bulk 0.35 eV (mid-infrared response). This layer-number dependence is further supported in Figure 1d. With increasing layer number, enhanced interlayer van der Waals interactions significantly reduce the bandgap and modify valence/conduction band curvatures, thereby tuning carrier effective masses and transport properties, providing a layer-degree-of-freedom for device performance regulation.

Figure 1e shows polarized absorption spectra of monolayer, bilayer, trilayer, and bulk BP along the armchair direction (incident absorbance vs. photon energy; x: armchair-polarized light; y: zigzag-polarized light). The absorption anisotropy is quantitatively presented in Figure 1g, where the optical absorption spectra of few-layer BP under two polarization directions (x and y) demonstrate distinct differences in intensity and peak number [22]. As shown in Figure 1g, in few-layer BP, both absorption intensity and characteristic peak number along the armchair direction (x) differ significantly from those along the zigzag direction (y), demonstrating strong polarization-dependent absorption. It has also been reported [23] that BP’s structural orientation can be determined by water droplet shape on its surface, facilitating anisotropy studies.

For few-layer BP, strong in-plane anisotropy is observed, manifested by strong light absorption in the x-direction and weak light absorption in the y-direction (Figure 1i). As layer number increases from monolayer to trilayer, characteristic excitonic absorption peaks (e.g., $E_{1}^{1}$, $E_{1}^{3}$) along the armchair direction continuously redshift to lower photon energies, reflecting bandgap reduction with increasing layers [22] and enabling layer-controlled band engineering. For bulk BP, sharp excitonic peaks disappear completely, the absorption curve flattens, and the absorption edge redshifts significantly to the low-energy region (corresponding to its 0.35 eV narrow bandgap). Meanwhile, in-plane anisotropy is greatly weakened, with absorption curves for the two polarization directions nearly overlapping, as interlayer coupling saturates and the quantum confinement effect vanishes entirely (Figure 1g).

The XRD pattern of 2D BP shows orthorhombic characteristic diffraction peaks (020), (040), and (060) [28] (Figure 1h). Its Raman spectrum exhibits three characteristic modes [23,25,29] (Figure 1e,f): $A_{g}^{1}$ (≈361 cm^−1^, lowest frequency, out-of-plane vibration perpendicular to the layer plane, leftmost peak), $B_{2g}$ (≈438 cm^−1^, intermediate frequency, in-plane vibration along the zigzag direction, middle peak), and $A_{g}^{2}$ (≈465 cm^−1^, highest frequency, in-plane stretching along the armchair direction, strongest rightmost peak). Raman spectroscopy can determine the exact layer number of as-prepared BP based on peak position and intensity differences (Figure 1e). Additionally, the ratio of the $A_{g}^{2}$ and $A_{g}^{1}$ Raman peak intensities exhibits strong polarization dependence (Figure 1i), providing a non-destructive method for determining BP’s crystal orientation without requiring transmission electron microscopy.

## 3. Controllable Preparation of Two-Dimensional Black Phosphorus

The well-established preparation methods of typical 2D van der Waals (vdW) materials (graphene, h-BN, TMDCs) have provided key guidance for developing 2D BP preparation techniques. Numerous BP preparation methods have been reported, and herein we systematically categorize them into three main categories based on their principal preparation objectives and application scenarios: small-batch high-quality device research, large-scale processing methods, and wafer-scale thin film growth [30].

### 3.1. Small-Batch Fabrication of High-Quality Devices

#### 3.1.1. Mechanical Exfoliation

Mechanical exfoliation is the most widely adopted top-down method for preparing 2D nanosheets from BP single crystals. It is valued for its simplicity, high crystallinity, and low contamination, and is regarded as a classic approach to obtain atomically flat, low-defect 2D layered materials [31]. Its effectiveness has been demonstrated by early studies that successfully isolated few-layer BP nanosheets, enabling the first proof-of-concept BP field-effect transistors [10,32]. However, the method has inherent limitations: it cannot achieve precise, batch-controllable fabrication, and the exposed surface is prone to degradation via reaction with water and oxygen (a point that will be addressed in Section 4.3). Consequently, despite yielding the highest-quality nanosheets, mechanical exfoliation remains unscalable and is limited to fundamental research and proof-of-concept devices.

#### 3.1.2. Chemical Vapor Transport

Chemical vapor transport (CVT) is the dominant method for producing high-quality bulk BP single crystals [33], the primary precursors for mechanical exfoliation of high-performance 2D BP nanosheets, with principal growth parameters including mineralizer system, raw material ratio, temperature gradient and heating/cooling program.

Extensive optimizations have improved 2D BP precursor quality: Zdeg et al. [26] achieved high-mobility crystals enabling record 2D FET performance; Fu et al. [34] synthesized doped precursors for stable 2D BP; Kuchkaev et al. [33] produced large high-purity crystals; Zhang et al. [35] clarified nucleation for reproducible 2D flakes; Wan et al. [36] demonstrated direct growth of large-area 2D BP nanosheets, marking a critical breakthrough in the field.

Despite these significant advances, conventional CVT methods still struggle to simultaneously achieve high crystal quality, high production yield, and large crystal size, which remains a major bottleneck for scalable manufacturing of 2D BP devices. To address this limitation, several innovative CVT variants have been developed, including bidirectional CVT [37] and an eco-friendly two-step heating CVT process [38]. Most notably, Chen et al. [39] proposed a continuous precursor release strategy by filling the middle section of the reactor with silica sand as a porous diffusion matrix. This design partitions the reaction chamber into a precursor zone and a growth zone, forcing P_4_ vapor to transport via a diffusion mode rather than the conventional convection mode, thereby establishing a quasi-static equilibrium in the growth region. This diffusion control significantly suppresses nucleation density (reducing the nucleation seed density to approximately 0.8 mm^−2^ when the diffusion section length is increased to 10 mm) and effectively promotes the layered lateral growth of single-crystal BP thin films. Ultimately, this method successfully produced millimeter to sub-centimeter scale thin films, confirming the critical role of the porous medium diffusion strategy in preparing high-quality 2D BP thin films.

Overall, CVT remains the most important method for producing high-quality 2D BP precursors. While direct growth of large-area, uniform 2D BP thin films is still an active area of research, the aforementioned innovations have brought us significantly closer to scalable production of BP-based electronic and optoelectronic devices. That said, it must be noted that CVT itself cannot directly yield thin films; it produces bulk precursors that still require subsequent exfoliation.

### 3.2. Large-Scale Processing Methods

#### 3.2.1. Liquid-Phase Exfoliation

Liquid-phase exfoliation overcomes the interlayer van der Waals forces of BP through physical, chemical, or electrochemical actions, exfoliating bulk BP into few-layer or monolayer nanosheets in a liquid medium. This method features mild reaction conditions, convenient operation, and large-scale production capability, making it an essential preparation route for the application of two-dimensional BP.

Solvent selection is critical to determining exfoliation efficiency and product quality. Solvents matching the surface energy of BP can lower the exfoliation energy barrier, helping to stabilize nanosheets and prevent agglomeration; the interaction between solvents and precursors directly affects the exfoliation efficiency, flake thickness, and size distribution of the products; in addition, organic solvents can effectively isolate oxygen and water, preventing oxidation of nanosheets during exfoliation [40]. Kuchkaev et al. [33] obtained BP nanosheets with thicknesses of 0.8–17 nm (approximately 1–30 layers) in N-methyl pyrrolidone (NMP) via ultrasonic liquid-phase exfoliation. However, although commonly used strong polar organic solvents such as NMP, dimethylformamide (DMF), and dimethyl sulfoxide (DMSO) can achieve effective intercalation and exfoliation, they generally suffer from high toxicity, poor environmental friendliness, and difficult removal of solvent residues [17,41]. To address these limitations, researchers have developed various solvent optimization strategies from different perspectives: some developed low-boiling-point solvents to avoid residue issues, some explored green organic solvents to reduce toxicity, and others proposed functionalized alkaline solvents to enhance the stability of exfoliated nanosheets [25,42,43].

Overall, liquid-phase exfoliation has a simple process flow and strong controllability, enabling efficient large-scale preparation in a short time [44], and is a main technology for promoting the macroscopic application of BP. However, this method still has inherent limitations: it is difficult to achieve precise control over layer number and size, the flake uniformity is much lower than that of mechanically exfoliated and CVD-grown samples, and defects and impurities are easily introduced during exfoliation. Consequently, while liquid-phase exfoliation struggles to meet the stringent requirements of high-performance devices, its attributes make it particularly well-suited for printed electronics, where scalability and low-cost processing are prioritized over ultimate device performance.

#### 3.2.2. Electrochemical Exfoliation

Electrochemical exfoliation is an important top-down wet chemical process for preparing 2D BP nanosheets. It utilizes an electric field to drive electrolyte ions to intercalate between BP layers, weakens interlayer van der Waals forces through volume expansion induced by ionic intercalation, and finally achieves efficient exfoliation. It is mainly divided into two routes: anodic and cathodic exfoliation. To improve the exfoliation efficiency and product quality, researchers have optimized the electrochemical process from multiple aspects, including intercalation ion regulation, electrolyte system modification, and potential control, which have enabled tunable layer number and scalable production of BP nanosheets.

In the anodic exfoliation route, Erande et al. [45] obtained few-layer BP nanosheets through the synergy of anionic intercalation and oxidation. Huang et al. [46] proposed a layer-tunable electrochemical cathodic exfoliation technology to address the problem of uncontrollable layer number, achieving layer number regulation by controlling tetraalkylammonium cation intercalation and applied voltage. Ambrosi et al. [47] realized simple aqueous electrochemical exfoliation in acidic aqueous solution, but the obtained BP had undergone oxidation and contained tin contamination. In the cathodic exfoliation route, Kuchkaev et al. [48] obtained BP nanosheets with a height of 8–10 nm and a lateral size of 0.8–1.0 μm via cathodic electrochemical exfoliation. Xiao et al. [49] designed a cathodic exfoliation method with low oxidation degree, using a solution of tetrabutylammonium hexafluorophosphate in propylene carbonate to obtain 2D BP with fewer layers. Relevant studies [46,50] have also confirmed the feasibility of cathodic exfoliation.

Collectively, these efforts have advanced electrochemical exfoliation from an empirical process toward a more controllable strategy, especially through intercalation ion engineering and cathodic low-oxidation designs. Yet the persistent trade-offs among oxidation degree, layer-number uniformity, and purity underscore that a truly defect-free, batch-consistent route remains an open challenge.

#### 3.2.3. High-Energy Ball Milling

High-energy ball milling is an important technical route for the large-scale preparation of BP nanomaterials. It integrates two primary functions: phase transformation synthesis of phosphorus allotropes and interlayer exfoliation of bulk BP. Complementary to small-batch high-precision preparation methods such as CVT and CVD, it is one of the key candidate processes for the large-scale application of BP. This method inputs high-intensity mechanical energy during the ball milling process to achieve crystal phase transformation between phosphorus allotropes, overcomes the interlayer van der Waals forces of BP to realize interlayer exfoliation, and finally obtains BP powder or two-dimensional BP nanosheets [51]. It has the fundamental advantages of simple process flow, low preparation cost, and high yield, but also has the disadvantages of many product defects, uneven flake size, and easy introduction of impurity contamination.

Direct phase transformation synthesis using red phosphorus as the raw material is the central application direction of high-energy ball milling for BP preparation. Relevant studies have shown that pressure plays a more important leading role than reaction temperature in the phase transformation synthesis process [52]. Shin et al. [53] used high-energy ball milling under a dry argon atmosphere to avoid oxidation and reverse transformation of BP, obtaining a large amount of micron-sized BP and a small amount of nanoscale BP. In addition to phase transformation synthesis, high-energy ball milling can also be used for the exfoliation of bulk BP to prepare two-dimensional BP nanosheets, providing a convenient process route for the preparation of BP composites. However, systematic and in-depth research on the influence of ball milling process parameters on the structure and properties of few-layer BP is still relatively lacking. In recent years, researchers have also developed various improved ball milling technologies to solve the defects of traditional processes, including plasma-assisted ball milling, vibratory ball milling, and planetary ball milling [52]. By controlling the magnitude of input mechanical energy, red phosphorus can be completely converted into BP without pollution and by-products.

Collectively, these innovations have advanced high-energy ball milling into a scalable, dual-function platform that integrates phase transformation and exfoliation, providing a low-cost and easily scalable solution for large-scale BP preparation and application. Yet, the persistent trade-off between high yield and defect control, the poor controllability of crystallization quality, and an insufficient systematic understanding of process-structure-property relationships confine its current outputs primarily to composites and bulk applications rather than high-performance electronic devices, underscoring the need for further process optimization.

### 3.3. Wafer-Scale Thin Film Growth

#### 3.3.1. Chemical Vapor Deposition

CVD achieves in-situ deposition through chemical reactions of gaseous precursors on the substrate surface, enabling large-area and thickness-controllable growth of 2D materials directly on target substrates. It is currently the most promising preparation technology for realizing device-oriented and large-scale applications of 2D BP thin films [54].

However, the controllable CVD preparation of 2D BP still faces intrinsic challenges: BP has a large specific surface area and poor environmental stability, making it extremely prone to oxidative decomposition at high temperatures, which imposes extremely stringent requirements on the system vacuum and inert atmosphere purity. Meanwhile, the high saturated vapor pressure of phosphorus sources makes it difficult to precisely control the diffusion behavior of gaseous phosphorus molecules, resulting in poor film quality, limited single-crystal size, and low batch-to-batch reproducibility—this is the principal bottleneck restricting the CVD preparation of 2D BP. To address the problems of uncontrollable phosphorus source diffusion and difficult substrate nucleation, researchers have made a series of breakthroughs starting from phosphorus source regulation and nucleation-assisted design. Xu et al. [54], based on the Au_3_SnP_7_-assisted BP nucleation mechanism, suppressed the excessive diffusion of phosphorus sources through multiple pathways and precisely controlled the temperature gradient, successfully growing millimeter-scale BP single-crystal thin films directly on silicon substrates with thickness precisely tunable from several nanometers to hundreds of nanometers. Field-effect transistors fabricated based on these films exhibited a room-temperature hole mobility exceeding 1200 cm^2^·V^−1^·s^−1^ and an on/off ratio of 10^6^, verifying the application potential of CVD-grown BP thin films in high-speed logic devices.

**Figure 2:**
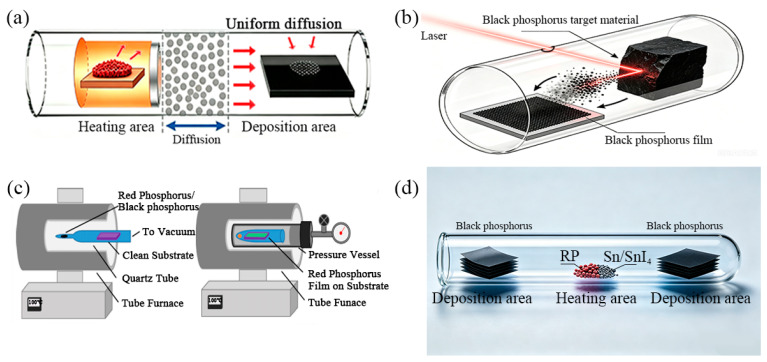
(**a**) Schematic diagram of phosphorus source diffusion modulation via porous materials [39]; (**b**) Panel showing the principle of PLD technology [56]; (**c**) Preparation of BP thin films via red phosphorus thin film deposition (**left**) followed by high-temperature and high-pressure treatment (**right**) [57]. Copyright 2021, Springer Nature; (**d**) Schematic diagram of the bidirectional CVT growth process [37].

On this basis, Cheng’s group [39] further developed a slow-release growth strategy of phosphorus sources based on porous materials, which utilizes silica sand as a chemically inert porous diffusion matrix to fundamentally change the transport mode of P_4_ precursor from uncontrolled forced convection to steady molecular diffusion. This porous matrix design significantly suppresses the violent fluctuation of P_4_ partial pressure in the growth chamber, establishing a quasi-static equilibrium growth environment that is critical for single-crystal growth. By precisely adjusting the length of the porous diffusion section, the nucleation density of BP can be quantitatively regulated: a 10 mm diffusion section reduces the nucleation density by three orders of magnitude to ~0.8 mm^−2^, which decouples the nucleation rate and edge growth rate of BP and makes the lateral layer-by-layer growth mode dominant. This strategy enabled the controllable preparation of sub-centimeter-scale BP single-crystal thin films. The obtained films had excellent crystallinity (with a rocking curve full width at half maximum (FWHM) of the (040) diffraction peak as low as 0.08° [39], comparable to bulk single crystals) and good electrical properties, further promoting the compatibility of BP thin films with silicon-based processes. In addition, innovations in precursor systems and catalytic substrates are also crucial directions for improving the quality and growth controllability of CVD-grown BP single crystals (Figure 2a).

These advances have yielded sub-centimeter BP single crystals with near-bulk crystallinity and high carrier mobility, highlighting CVD as a vital route for device-oriented 2D BP thin films; however, the intrinsic high-temperature instability, limited scalability to wafer scale, and silicon compatibility issues remain fundamental barriers, calling for transformative strategies inspired by mature TMDC growth technologies to achieve true wafer-scale, silicon-compatible BP.

#### 3.3.2. Pulsed Laser Deposition

PLD belongs to the category of physical vapor deposition (PVD) technologies and is an important technical route for the controllable preparation of two-dimensional thin film materials. Currently, there are few reports on the controllable preparation of two-dimensional BP by PLD. Existing studies mostly focus on phosphorus-doped thin films, whose deposition principles are similar to those of BP thin films—only replacing the target with high-purity phosphorus is required to achieve BP thin film preparation. Yang et al. [55] achieved the growth of BP thin films at a low temperature of 150 °C. Using graphene/Cu or SiO_2_/Si as substrates with a target-substrate distance of 4 cm, they ablated a BP target with a 248 nm wavelength pulsed laser and obtained two-dimensional BP with a lateral size of 4.9 nm and a thickness of 1.9 nm, verifying the feasibility of low-temperature BP preparation by the PLD method. Wu et al. [56] further developed a PLD process for the controllable growth of centimeter-scale high-quality few-layer BP on this basis. With a growth temperature of 300 °C followed by rapid cooling, the fabricated field-effect transistors exhibited good performance, breaking through the size limitation of BP preparation by the PLD method (Figure 2b).

Overall, PLD shows exceptional potential for growing large area high quality BP thin films, providing a basis for electronic, optoelectronic devices and semiconductor integrated circuits based on BP. Table 1 presents an objective summary of the application scopes, advantages, and limitations of the aforementioned preparation methods. CVD and PLD show great potential for large-scale applications, while other methods have their own strengths and weaknesses. Researchers should choose appropriate methods according to experimental needs.

### 3.4. Other Preparation Methods

In addition to the mainstream technologies, several supplementary routes exist for two-dimensional (2D) BP preparation, with bulk crystal synthesis methods only serving as precursor backgrounds.

Early bulk BP precursor methods include the high-temperature high-pressure approach by Shirotani [58], which enables rapid synthesis of high-purity crystalline bulk crystals but suffers from stringent equipment requirements and high safety risks. The liquid-phase recrystallization method by Brown et al. [59] offers milder conditions but severe solvent residual contamination limits its application as a 2D BP precursor.

For direct 2D BP fabrication, Zheng et al. [60] developed a pulsed laser-assisted liquid-phase exfoliation method to control the thickness and size of 2D BP nanosheets. Another indirect route converts pre-deposited red phosphorus thin films into BP [57] (Figure 2c), avoiding uncontrollable phosphorus source diffusion in direct vapor-phase growth, though its crystallization quality and conversion efficiency still need improvement and it requires high-pressure conditions.

Overall, these methods provide technical supplements but cannot replace the mainstream CVT-mechanical exfoliation route for laboratory 2D BP research, while CVD and PLD remain the essential directions for future wafer-scale 2D BP production.

### 3.5. Synthesis Summary of 2D Black Phosphorus

In summary, PLD shows exceptional potential for growing large area high quality BP thin films, providing a basis for electronic, optoelectronic devices and semiconductor integrated circuits based on BP.

Table 1 presents an objective summary of the application scopes, advantages, and limitations of the aforementioned preparation methods. To further evaluate their industrialization potential, we conduct a critical comparative analysis from three core dimensions: scalability, production cost, and product quality. In terms of scalability, mechanical exfoliation is inherently limited to laboratory-scale single-sample preparation, while liquid-phase exfoliation, electrochemical exfoliation and high-energy ball milling enable kilogram-scale production but suffer from poor batch consistency. CVT can produce centimeter-sized bulk crystals but cannot directly fabricate thin films, requiring subsequent exfoliation steps that hinder continuous manufacturing. In contrast, CVD and PLD have demonstrated centimeter-scale uniform thin film growth, showing the clearest path to wafer-scale production. From a cost perspective, high-energy ball milling is the most cost-effective route with low equipment and raw material costs, followed by liquid-phase exfoliation. CVT has moderate costs, while mechanical exfoliation and PLD currently have higher scaling-up costs due to low throughput and high equipment investment; however, PLD costs are expected to decrease significantly with the development of high-power laser systems and batch processing technologies. Regarding product quality, mechanically exfoliated nanosheets remain the gold standard with nearly defect-free crystal structures and record carrier mobility. CVD-grown thin films have achieved near-bulk crystallinity (rocking curve FWHM as low as 0.08°) and mobility exceeding 1200 cm^2^·V^−1^·s^−1^, approaching the performance of exfoliated samples. PLD produces moderate-quality films with lower defect density than solution-based methods, while CVT provides the highest-quality bulk precursors. Solution-based methods generally yield products with high defect densities and broad size distributions, limiting their use in high-performance devices.

Based on this comprehensive comparison, CVD and PLD are the most promising routes for industrial applications of 2D BP. CVD offers the best balance between scalability and product quality, with the ability to directly grow high-mobility thin films on silicon substrates, making it ideal for large-scale logic and optoelectronic devices. PLD’s unique advantage of low-temperature growth (150–300 °C) enables monolithic integration with existing silicon CMOS processes without damaging pre-fabricated circuits, which is critical for practical semiconductor manufacturing. While other methods will continue to play important roles in specific scenarios (e.g., mechanical exfoliation for fundamental research, ball milling for composite materials), overcoming wafer-scale uniformity control and process cost reduction bottlenecks for CVD and PLD will be the key to realizing commercialization of 2D BP-based devices.

## 4. Applications of Two-Dimensional Black Phosphorus

BP features a high hole mobility, widely tunable direct bandgap, and excellent on/off current ratio, which renders it highly promising for applications in various fields including optoelectronic devices (e.g., photodetectors) and electronic devices (e.g., FETs).

### 4.1. 2D Black Phosphorus FET

High mobility enables fast carrier transport in BP, which directly translates to higher operating frequency and switching speed of transistors, making it an ideal channel material for high-performance electronic devices.

**Figure 3:**
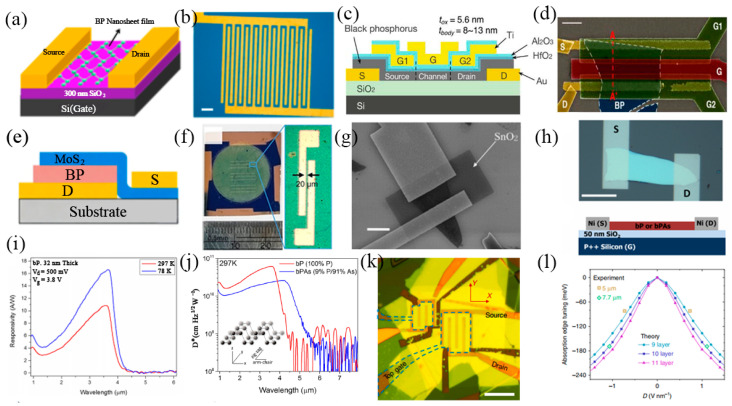
(**a**) 2D BP field-effect transistor (FET) [45]. Copyright 2016, American Chemical Society; (**b**) Optical image of the as-fabricated FET, scale bar is 20 μm [45]. Copyright 2016, American Chemical Society; (**c**) Schematic of the BP RED-TFET [61]. Copyright 2019, American Chemical Society; (**d**) Representative false-colored SEM image of the BP RED-TFET. Scale bar: 1 μm [61]. Copyright 2019, American Chemical Society; (**e**) Device structure of a BP mid-infrared photodetector [62]. Copyright 2024, American Chemical Society; (**f**) 2D BP transistor fabricated by electrochemical exfoliation [63]. Copyright 2022, Jeon(s), used under CC BY 4.0; (**g**) Scanning electron microscopy (SEM) image of an as-fabricated BP FET covered with a patterned SnO_2_ thin film, Scale bar is 2 μm [64]. Copyright 2019, Science Bulletin; (**h**) Optical microscope image (scale bar, 10 μm) and cross-sectional schematic of a typical 29 nm thick BP gated photoconductor [65]. Copyright 2017, American Chemical Society; (**i**) Broadband spectral response of BP-As photodetectors [65]. Copyright 2017, American Chemical Society; (**j**) Specific detectivity (D*) of BP and BP-As photoconductors, [65]. Copyright 2017, American Chemical Society; (**k**) Structure of the tunable BP mid-IR photodetector based on a dual gate transistor configuration [27], used under CC BY 4.0; (**l**) Modulation of the optical absorption edge in 10-layer BP under bias [27], used under CC BY 4.0.

In 2014, Li et al. [10] demonstrated the first p-type BP FET fabricated via high-temperature high-pressure synthesis combined with mechanical exfoliation. The device showed a drain current modulation of ~10^5^ and a room-temperature hole mobility of ~1000 cm^2^·V^−1^·s^−1^, which for the first time verified the great potential of BP in transistor applications and laid the foundation for all subsequent research. Representative device schematics and optical images of BP FETs are shown in Figure 3a,b,f.

Subsequent studies have focused on improving the electrical performance of BP FETs through two main approaches: surface modification and preparation process optimization. For instance, Guo et al. [66] developed a metal ion modification strategy, where silver ion doping enhanced the hole mobility by more than 2-fold and increased the on/off current ratio by 44-fold. BP FETs fabricated from CVT-grown materials also exhibited excellent performance: thin-film-based devices showed good electrical characteristics [36], while single-crystal-based devices achieved a record high mobility and excellent switching performance [38]. Beyond performance optimization, researchers have also explored the fundamental device physics of BP FETs and their integration potential. Liu et al. [67] revealed that the mobility of BP transistors exhibits a strong thickness dependence, reaching a maximum at 5 nm, and clarified the correlation between carrier transport anisotropy and the armchair crystal orientation. Based on these findings, they successfully constructed a complementary metal-oxide-semiconductor (CMOS) inverter by integrating p-type BP MOSFETs with n-type MoS_2_ MOSFETs, realizing the heterogeneous integration of BP and providing a promising solution for future electronic systems based on 2D semiconductors.

Wu et al. successfully developed a few-layer BP reconfigurable electrostatically doped tunneling field-effect transistor (BP RED-TFET) with a triple-top-gate structure (schematic in Figure 3c, top-view in Figure 3d) [61]. The BP channel had an initial thickness of 8–13 nm, with the effective channel thickness slightly reduced due to surface oxidation. The gate oxide thickness was approximately 5.6 nm, and the overall device was on the micrometer scale. By leveraging multi-gate control for electrostatic doping of the source and drain regions, the device could be flexibly switched among four operating modes: n-type tunneling FET (TFET), p-type TFET, and conventional n-/p-type MOSFETs. At room temperature, the device achieved an on-current of 0.6 μA/μm and a subthreshold swing of 170 mV/dec. Combining microstructural characterization with atomistic quantum transport simulations, they further predicted that by scaling the equivalent oxide thickness to 0.5 nm and adopting monolayer BP as the channel, the on-current could be boosted to 800 μA/μm with a subthreshold swing as low as 12 mV/dec. This work convincingly demonstrates the application value of BP in high-performance, low-power tunneling devices and provides crucial insights for the design and optimization of energy-efficient transistors based on 2D materials [61].

Despite these impressive laboratory achievements, practical industrial deployment still faces three core challenges: large-scale manufacturing remains hindered by poor wafer-scale uniformity and unavoidable defects introduced during the transfer process, leading to significant device-to-device variation; long-term device reliability is compromised by ambient-induced degradation and threshold voltage drift under continuous operation; and compatibility with standard silicon CMOS technology remains limited due to mismatched growth temperatures and high contact resistance at the BP-silicon interface.

### 4.2. 2D Black Phosphorus Photodetector

Two-dimensional black phosphorus (BP) has emerged as a highly promising material for optoelectronic devices, particularly photodetectors, owing to its exceptional electronic and optical properties. Its layer-tunable direct bandgap, ranging from 0.3 eV in bulk form to 1.7 eV in the monolayer limit, enables broad-spectrum photoresponse spanning the visible to mid- and far-infrared regions, endowing it with irreplaceable advantages in broadband detection, and the structural diagram of a BP mid-infrared photodetector is displayed in Figure 3e. Complementing this broadband capability is BP’s high carrier mobility, which ensures efficient photogenerated carrier collection and fast response speed. To further enhance photodetection performance, BP-based photodetectors predominantly adopt van der Waals heterojunction designs, with the p-type BP/n-type MoS_2_ heterojunction being the most representative. This architecture effectively suppresses dark current while promoting photogenerated carrier separation and transport, thereby significantly improving overall optoelectronic performance.

Researchers have leveraged these unique properties to achieve remarkable breakthroughs in BP photodetector performance across multiple critical dimensions. In terms of broadband detection capability, Amani et al. [65] demonstrated a typical 29 nm thick BP gated photoconductor shown in Figure 3h; pure BP photodetectors cover a detection range from 1 μm to 3.5 μm, while BP-As alloys extend the composition-tunable cutoff wavelength to 3.8–4.6 μm, and their broadband spectral response curves are presented in Figure 3i. The room-temperature specific detectivities reached up to 6 × 10^10^ cm·Hz^1/2^·W^−1^ for pure BP and 2.4 × 10^10^ cm·Hz^1/2^·W^−1^ for BP-As (91% As) photoconductors, as summarized in Figure 3j. Building on the widely adopted van der Waals heterojunction strategy, Zhu et al. [68] developed a non-volatile MoS_2_/BP heterojunction infrared detector that integrates photodetection, memory, and computing functionalities in a single device. This device exhibits a response peak at 3.6 μm and a cutoff wavelength of approximately 3.9 μm, representing a typical mid-infrared photodetector with multifunctional integration capabilities. Beyond these material composition and heterojunction engineering approaches, Chen et al. [27] proposed a tunable BP mid-infrared photodetector based on a dual gate transistor configuration (Figure 3k). A vertical displacement field can dynamically modulate the optical absorption edge of 10-layer BP via the Stark effect, redshifting the photoresponse cutoff from 3.7 μm to beyond 7.7 μm, and the corresponding regulation result is shown in Figure 3l, which is in excellent agreement with theoretical calculations. For sensitivity and response speed, Shen et al. [69] developed an asymmetric Schottky BP transistor that delivered high responsivity and detectivity in the near-infrared communication band, while Zhang et al. [70] designed a vertical-structure BP photodiode that realized ultrafast response in the mid-infrared region, greatly expanding BP’s application scope in high-speed infrared detection. Additionally, BP’s pronounced in-plane anisotropy endows it with inherent polarization-dependent light absorption, providing a unique degree of freedom for polarization-selective photodetectors. Yuan et al. [71] constructed a linear birefringence photodetector that achieved polarization-sensitive response across an ultra-broad spectral range, and Bullock et al. [13] further realized monolithically integrated polarization-resolved photodetectors, advancing the practical application of BP polarization detection technology.

Despite these remarkable performance breakthroughs in laboratory settings, translating BP photodetectors into practical imaging and sensing systems faces three interconnected fundamental challenges. First, core performance bottlenecks persist in large-area devices: high dark current originating from surface defects and interface states severely limits detection sensitivity at room temperature, while non-uniform layer thickness and transfer-induced defects lead to significant pixel-to-pixel photoresponse variation and low fabrication yield for focal plane arrays. Second, device reliability remains a critical concern: photo-induced degradation under prolonged infrared illumination accelerates surface oxidation, causing irreversible increases in dark current and continuous decay of responsivity, which cannot meet the long-term stability requirements of commercial optoelectronic systems. Third, silicon integration compatibility issues hinder system-level implementation: current hybrid integration schemes require separate fabrication and transfer of BP devices, increasing system complexity, power consumption and packaging cost, while direct monolithic integration is limited by mismatched growth temperatures and interface contamination that degrade both BP and pre-fabricated silicon circuit performance. Overall, 2D BP exhibits tremendous potential in optoelectronic devices, particularly in mid-infrared and polarization-sensitive detection. Table 2 summarizes the critical metrics of graphene, BP and MoS_2_, providing a direct comparison of their fundamental properties and application potentials.

### 4.3. Strategies for Blocking the Degradation of Black Phosphorus

Ambient instability remains the most critical bottleneck restricting the practical application of black phosphorus (BP)-based devices. Zhou et al. [78] clarified the key three-step mechanism of BP environmental degradation via computer simulation: under illumination, oxygen reacts with surface lone pair electrons to form oxygen ions, which then generate phosphorus oxides that subsequently decompose in the presence of water. They also found that complete surface oxidation forming P-O-P bonds can effectively suppress performance degradation. However, surface lone pair electrons of phosphorus atoms are still the fundamental cause of BP instability, which has guided the rational design of targeted stabilization strategies.

The most mainstream direction of BP stabilization modification is to isolate BP from contact with water and oxygen in the environment through coating, encapsulation and other methods to block the environmental inducement of the degradation reaction. Such strategies can simultaneously realize the regulation and optimization of BP device performance. For example, the BP interlayer heterojunction prepared by Chen et al. [79] not only ensured the long-term stability of BP under ambient conditions, but also achieved a high-quality on/off ratio of more than 10^5^ and a room-temperature mobility of 1350 cm^2^·V^−1^·s^−1^, achieving an excellent balance between environmental stability and device performance. Similarly targeting the critical issue of ambient instability, Wu et al. [64] proposed an ultrathin SnO_2_ passivation strategy, which significantly improved the atmospheric stability of BP devices without compromising their electrical performance, enabling long-term stable operation in air (Figure 3g).

Moving beyond physical isolation approaches, researchers have developed a series of strategies that target the root cause of BP instability: the reactive surface lone pair electrons. Zhao et al. [40] designed titanium benzenesulfonate ligands, which block the reaction between lone pair electrons and oxygen through coordination interactions, thereby inhibiting oxidative degradation at the source. Guo et al. [66] found that pure BP is extremely susceptible to oxidative degradation by oxygen and water vapor in an air environment with 95% relative humidity, with devices almost completely failing after 24 h. In contrast, silver ions can spontaneously adsorb on the BP surface via cation-π interactions to passivate the lone pair electrons of phosphorus atoms, effectively blocking the oxidation pathway. The modified BP flakes remained stable in high-humidity air for more than 5 days, and the electrical performance of the corresponding transistors was maintained for nearly 3 days; furthermore, the devices exhibited robust stability under both high and low temperature conditions. This work successfully proposed a simple yet effective metal ion modification strategy that simultaneously enhanced the environmental stability of BP and the electrical performance of BP-based transistors. This method is also generalizable to other metal ions including Fe^3+^, Mg^2+^ and Hg^2+^, providing an important approach for the development and practical application of highly stable, high-performance BP-based electronic and optoelectronic devices.

In summary, ambient instability remains the most critical bottleneck restricting the practical application of BP-based devices. To address this challenge, researchers have developed two core mitigation strategies. The first is the mainstream physical isolation approach, which employs coating or encapsulation to sever contact with environmental water and oxygen, while simultaneously enabling the regulation and optimization of device performance—exemplified by interlayer heterojunctions and ultrathin SnO_2_ passivation techniques. The second is surface lone pair passivation, which targets the root cause of instability by blocking oxidative degradation pathways at the source via coordination interactions or cation-π interactions. Metal ion modification strategies, particularly silver ion (Ag^+^) decoration, stand out for their simplicity, high efficiency, and generality, as they concurrently enhance both the environmental stability of BP and the electrical performance of the devices.

## 5. Conclusions

Two-dimensional black phosphorus (2D BP) has emerged as a transformative layered semiconductor for next-generation electronics and optoelectronics, distinguished by its unique layer-dependent direct bandgap spanning ~0.3 eV (bulk) to ~1.7 eV (monolayer) covering the visible to mid-infrared spectrum, room-temperature hole mobility exceeding 1200 cm^2^·V^−1^·s^−1^, and strong in-plane anisotropy enabling polarization-sensitive devices. These characteristics address critical limitations of graphene (zero bandgap) and TMDCs (poor mid-infrared absorption), establishing BP as an indispensable complementary 2D semiconductor. This review systematically categorizes its preparation advances by application scenarios: optimized CVT remains dominant for high-quality bulk precursors, with breakthroughs in centimeter-sized single crystals via bidirectional CVT [37] and direct large-area 2D BP growth [36]; liquid-phase exfoliation, electrochemical exfoliation and high-energy ball milling enable scalable solution processing; while CVD has achieved millimeter-scale single-crystal films on silicon [54] and PLD enables low-temperature (150–300 °C) centimeter-scale growth [56], showing great potential for wafer-scale production.

These preparation innovations have driven significant device progress: the first BP FET demonstrated ~1000 cm^2^·V^−1^·s^−1^ mobility in 2014 [10], and subsequent metal ion modification enhanced mobility by 2-fold and on/off ratio by 44-fold [66], with BP/MoS_2_ complementary inverters successfully validating its logic circuit potential. For photodetectors, BP enables broadband detection from visible to mid-infrared, with breakthroughs including ultrafast nanosecond-response vertical photodiodes [70] and monolithically integrated polarization-resolved detectors [13], while ultrathin SnO_2_ passivation has achieved long-term ambient stability [64]. Nevertheless, three fundamental bottlenecks still hinder industrial application: intrinsic light-induced degradation via water-oxygen reaction, lack of wafer-scale single-crystal films with uniform layer thickness and unavoidable transfer-induced defects, and poor compatibility with mainstream silicon CMOS technology due to high growth temperatures and interface mismatch.

Future research efforts should focus on three principal directions: developing in-situ ligand passivation and interface engineering techniques to fundamentally address degradation by targeting reactive surface lone pair electrons; deepening the understanding of nucleation and growth mechanisms to realize precise layer-number control and wafer-scale single-crystal growth; and exploring low-temperature growth and transfer-free fabrication methods to achieve seamless monolithic integration with silicon platforms. Addressing these challenges will unlock the full potential of 2D BP in mid-infrared photonics, high-speed low-power electronics, and flexible wearable devices.

## Figures and Tables

**Table 1:** Comparison of preparation methods.

Method	Layer Control and Size	Crystal Quality	Key Advantage	Main Limitation
Mechanical exfoliation	Poor (random), ~10–100 μm	Highest	Nearly defect-free	Not scalable
Liquid-phase exfoliation	Moderate (after sorting), 100 nm–5 μm	Low	Scalable solution processing	Broad size distribution, residual solvent
Electrochemical exfoliation	Moderate, 0.5–10 μm	Moderate	Fast, mild conditions	Metallic contamination, oxidation
Chemical vapor transport (CVT)	N/A (bulk), mm-cm (crystals)	Very high	Fast, mild conditions	Not direct thin film
High-Energy Ball Milling	~0.1–10 μm	Lowest	Scalable, low cost, no high-pressure equipment required	Defects, broad size distribution
Chemical vapor deposition (CVD)	Good (thickness tunable), 100 μm-cm (film)	Good to excellent	Wafer-scale potential	High-temperature oxidation
Pulsed laser deposition (PLD)	Good, cm-scale film	Moderate	Low-temperature growth (150–300 °C)	High cost; limited area uniformity

**Table 2:** Comparison of key properties of graphene, black phosphorus, and MoS_2_.

Project	Graphene	BP	MoS_2_
Crystal structure	Sp^2^ honeycomb lattice; vdW layers.	Orthorhombic puckered lattice; anisotropic; vdW layered.	2H-phase trigonal prismatic; vdW layered.
Bandgap (monolayer/multilayer/bulk)	Monolayer: 0 eV	Monolayer: ~1.7 eV	Monolayer: ~1.8–1.9 eV
	Bilayer: intrinsic 0 eV	Bilayer: ~1.1 eV	Bilayer: ~1.5–1.6 eV
	Bulk: band overlap	Bulk: ~0.3 eV	Bulk: ~1.2–1.3 eV
Carrier mobility (ambient temperature)	~10,000 cm^2^·V^−1^·s^−1^	~1000 cm^2^·V^−1^·s^−1^	~140 cm^2^·V^−1^·s^−1^
On/off ratio	Band-modulated on/off ratio, BP/Graphene junction ~10^3^	~10^5^	~10^9^
Photoresponse wavelength range	UV to far-IR, but with extremely low responsivity	~600–3700 nm; electric field modulation: up to 7700 nm	~400–1000 nm
Air stability	Excellent	Poor	Excellent
References	[72,73]	[10,13,21,27]	[74,75,76,77]

## Data Availability

No new data were created or analyzed in this study. Data sharing is not applicable to this article.

## Abbreviations

The following abbreviations are used in this manuscript:

2D	Two-Dimensional
BP	Black Phosphorus
CVD	Chemical Vapor Deposition
CVT	Chemical Vapor Transport
FET	Field Effect Transistor
FWHM	Full Width at Half Maximum
NMP	N-Methyl Pyrrolidone
PLD	Pulsed Laser Deposition
TMDCs	Transition Metal Dichalcogenides
vdW	van der Waals
XRD	X-Ray Diffraction
